# 

**Published:** 1860-02

**Authors:** 


### CONTENTS FOR FEBRUARY, 1860.

### ORIGINAL COMMUNICATIONS.

A Critical Lecture on the Extirpation of the Parotid Gland, and its liability to malignant diseases. By TITUS DEVILLE, M. D., Prof. of Anatomy in the Medical Department of the Lind University; Associate of King's College, London, and late of the Ecole Pratique of Paris etc.... 65

Notes on Surgical Cases. By E. ANDREWS, M. D.... 90

Notes upon Diptheria. By J. H. HOLLISTER, M. D.,.... 94

Proceedings Chicago Academy of Medical Sciences.... 96

Abstract of Proceedings of Chicago Medical Society.... 100

### BOOK AND PAMPHLET NOTICES.

Introductory Address on Anatomy. By TITUS DEVILLE, M. D.... 110

Botany as an Ally of Medicine. A Lecture delivered to the Medical Society of the University of Nashville. By Geo. S. BLACKIE, M. D., T. B. S. E , etc.... 113

Miscellaneous Notices.... 115

### CLINICAL REPORTS.

Medical Wards of the Mercy Hospital. By Prof. DAVIS, M. D.... 116

Clinique of Prof. DAVIS, in the Medical Department of Lind University.... 121

### EDITORIAL.

The "Examiner" independent of all Schools.... 124

Medical Instruction in Chicago.... 124

Experimental Physiology.... 125

Prof. DEVILLE's Lecture.... 126

Rush Medical College Clinique.... 126

Chicago College of Pharmacy.... 127

Illinois State Medical Society.... 127

Flora of Illinois.... 128

A Favor.... 128

